# The National Tuberculosis Control Programme of Liberia Laboratory Programme Performance

**DOI:** 10.1155/2019/5340263

**Published:** 2019-07-07

**Authors:** Kassaye Tekie Desta, T. E. Masango, Zerish Zethu Nkosi

**Affiliations:** Department of Health Studies, University of South Africa, Pretoria, South Africa

## Abstract

**Background:**

Tuberculosis (TB) is a major public health problem in Liberia. Little is known about the TB laboratory performance of Liberia and the challenges after the 14 years of civil war which ended in 2003. The purpose of the study was to evaluate the TB laboratory performance of Liberia.

**Methods:**

A cross-sectional study was conducted from 2014 to 2015. The study was conducted using quantitative data of TB case findings, sputum microscopy proficiency testing, and on-site assessment of sputum microscopy laboratories in Liberia. 80 laboratories participated in the proficiency testing. Besides, four years' (2012–2015) TB case finding data obtained from the National Leprosy and Tuberculosis Control Programme (NLTCP) were used to complement the study. The data were analysed using descriptive statistics.

**Results:**

From the 80 TB sputum microscopy testing laboratories participating in proficiency testing, only 20 (25%) scored acceptable performance. 46 (58%) TB microscopy laboratories reported quantification errors for the proficiency panel slide 6 which was 3+. The national TB smear-positive cases notified were 4342 in 2012 but decreased to 3820 and 2448 in 2013 and 2014, respectively. The TB smear case detection rate showed an increase from 68% in 2010 to 78% in 2011 and a decrease to 60%, 57%, and 42% in 2012, 2013, and 2014, respectively.

**Conclusion:**

Between 2010 and 2013, the NLTCP succeeded in increasing the number of TB sputum microscopy laboratories. At most of the TB microscopy sites, the TB laboratory quality system was not implemented. The NLTCP of Liberia should develop strategies to overcome its challenges in TB laboratory testing.

## 1. Background

Liberia, Nigeria, and Sierra Leone are among the high TB burden countries in Africa. The prevalence of TB was 330, 307, and 308 per 100,000 population for the year 2014 in Nigeria, Sierra Leone, and Liberia, respectively [1]. The National Leprosy and Tuberculosis Control Programme (NLTCP) of Liberia was well functioning starting from its establishment in 1989 under the Department of Preventive Health Services of the Ministry of Health. However, the NLTCP was crippled by the 14 years of civil war which ended in 2003 [2]. The number of all forms of TB cases detected between 2005 and 2007 was as low as 49%. The TB programme services were significantly expanded thanks to the financial support obtained from the Global Fund to Fight AIDS, Tuberculosis and Malaria. As a result of this, the TB notification showed an increment between the years 2008 and 2012. The timely release of funding from the Global Fund resulted in a TB smear detection rate of 78% in 2011. There was a drop in the TB smear detection rate in 2012 as a result of stock-out of TB microscopy reagents in many facilities. The treatment success rate was 76% in 2012 [3, 4]. This figure is by far less than the national and global targets of 85% set by the Stop TB strategy of the WHO which ended in 2015 [5].

Active TB is diagnosed by chest X-rays, sputum smear microscopy examination, and microbiological culture specimens. Microscopy and chest radiography are the two widely used TB diagnostic tools in developing countries. The main limitation of these two TB diagnostic methods is the very low sensitivity and specificity which vary from 20% to 80% depending on the accuracy of the specimens collected [6, 7]. Children and individuals who are human immunodeficiency virus (HIV) coinfected have reduced pulmonary bacillary loads. As a result of this, the TB smear microscopy sensitivity is very low in these patients [6]. Lack of adequate space for smear microscopy testing, poorly maintained microscopes, lack of qualified TB microscopists, continuous stock-out of reagents, and lack of uninterrupted electricity and water are some of the challenges of TB smear microscopy in resource-limited countries [7].

In developing countries, TB cultures are not usually used to confirm TB diagnosis by smear microscopy. The capacity for drug susceptibility testing (DST) is very limited. TB cultures, compared to smear microscopy, are more expensive. TB cultures require high safety standards, specialised laboratory equipment, well-trained manpower, and an uninterrupted supply of electricity. Because of these, developing countries are limited to sputum microscopy only. TB culture laboratories in resource-poor countries are only limited at the national reference laboratories. However, quality assurance programmes including external quality assessment (EQA) are not implemented in most developing countries. Networking of TB local laboratories that provide acid-fast bacilli (AFB) plays a crucial role in the effective control of TB. This networking will assist in the successful expansion of directly observed treatment, short-course (DOTS), management of drug resistance TB, and HIV-associated TB [8]. This can be achieved by implementing a comprehensive EQA programme with sufficient administrative support and attention from the national TB control programmes [9]. In many countries, AFB microscopy is integrated into general clinical services. To achieve these goals, AFB smear procedure should be performed appropriately [8, 9]. The most effective strategy to assess the performance of TB laboratory diagnosis is by implementing EQA.

EQA is the best tool to compare and evaluate the performance TB microscopy laboratories in the network. EQA helps in identifying any deficiencies that may occur in participating laboratories and overcoming the gaps by implementing corrective actions. Panel testing, blinded rechecking, and on-site supervision are the three EQA schemes employed in national TB programmes [9, 10]. EQA is also an important means of motivation to the staff of the participating laboratories. In proficiency panel testing, panels are prepared, evaluated, and validated by the proficiency panel providing body and distributed to participating laboratories. Blind rechecking involves the collection of stained and stored TB slides for re-evaluation by qualified professionals from the regional or reference laboratories. The other EQA scheme, which involves visits to the peripheral laboratories by trained laboratory personnel from the reference or intermediate laboratory, is on-site evaluation. This scheme is purposefully designed to supervise the condition of equipment, laboratory safety, adequacy of supplies, the availability of SOPS, and electricity. The three EAQ schemes described above should be conducted regularly with timely corrective measures. This will significantly improve the performance of the TB microscopy testing [8].

In addition to the evaluation of the EQA scheme of TB laboratories of a national TB control programme, the national trends in TB case detection and the number of positive TB smear tests conducted per year in a country are good indicators of national TB laboratory performance. The TB case detection rate is the number of new smear-positive TB cases detected per estimated number of new smear-positive TB cases countrywide in one year. TB case detection of 70% was set as a national TB programme target by the STOP TB strategy of the WHO until the year 2015 [5].

The performance of the NLTCP of Liberia was critically affected by the 14 years of civil war which ended in 2003. The civil war crippled the entire health system—the health facility infrastructure, the financial support, and the healthcare workforce. At the end of the civil war, the Global Fund provided financial support and the NLTCP initiated its basic TB services [11]. Despite the effort of the NLTCP of Liberia in increasing the microscopy sites in the country, the quality of TB laboratory testing was poor. The national reference TB culture and DST laboratory was not fully functional for years. The performance of the TB laboratory programme of Liberia was not evaluated since the end of the civil war, and little is known about its coverage and the quality of service it provides. The objective of our study was to evaluate the performance of the national TB laboratory programme and provide recommendations based on the study findings.

## 2. Materials and Methods

A cross-sectional study was conducted using quantitative data obtained from proficiency testing and on-site supervision data. In addition to this, four years' (2012–2015) TB laboratory case finding data of the NLTCP of Liberia were used to evaluate the performance of the TB laboratory programme of Liberia. As part of the assessment of the NLTCP laboratory performance, 80 TB microscopy testing laboratories were included in the proficiency panel testing. TB sputum leftover specimens were collected anonymously from the TB annex hospital. TB slides were prepared from the leftover specimens at the National TB Reference Laboratory of Liberia and were distributed to facilities for testing them as part of their routine TB smear microscopy testing. Validated TB sputum-stained and TB sputum-unstained slides were distributed to the 107 functioning TB microscopy laboratories, and 80 of them were included in the proficiency panel assessment and results were returned using the reporting format. Each panel set consisted of 10 known smears: 5 stained (Ziehl–Neelsen staining) and 5 unstained. Both stained and unstained TB sputum slides consisted of negative and positive slides of different grades with code numbers of slide 1 to slide 10. Each slide carried 10 points, and the total maximum score was 100 (for 10 slides). A performance score of less than 90 (90%) was considered to be unacceptable by the WHO TB EQA standard [4, 8, 9]. In addition to this, on-site assessment of 107 functional TB microscopy laboratories was included in the study. However, only 93 laboratories were visited and included in the assessment. The study on the laboratory performance was done as part of EQA schemes using a standardised checklist.

### 2.1. Study Participants

Of the functional TB microscopy centres in Liberia, all 107 were included. Out of the 107 TB testing laboratories, 80 (75%) laboratories participated in the proficiency testing and 93 (87%) participated in the on-site assessment.

### 2.2. Data Collection and Analysis

Proficiency testing data from participating laboratories were collected by the 15 county diagnostic supervisors of the 15 counties and the NLTCP TB coordinators. TB programme laboratory data for the case detection rate and for the TB notified cases were collected by the principal investigator using self-developed and pretested checklists. Records and TB programme document reviews were used to collect the required data for the four years' TB case findings. An on-site assessment checklist was also developed to capture the TB laboratory performance from the 93 sites. The checklists were developed by reviewing a selection of literature. Data were entered into Excel for ease of statistical analysis. Descriptive statistics were used to analyse and summarise the collected data.

### 2.3. Ethical Considerations

The study was approved by the Higher Degree Committee of the University of South Africa (HSHDC/297/2014) and the National Ethics Board of Liberia (Ref.: NREB-008-16). Both institutions provided clearance certificates. The NLTCP of Liberia also granted permission to conduct the study following the ethical certificate. All assessment results were securely stored. Names of participating TB laboratories and results of their proficiency panel tests were not linked, and information was not provided to a third party without the knowledge of the Ministry of Health of Liberia.

## 3. Results

### 3.1. Results of TB Laboratories' Participation in TB Microscopy Proficiency Panel Testing

As part of the EQA of the TB microscopy laboratories, 80 facilities participated in the proficiency panel testing out of the 107 microscopy sites in Liberia. The TB proficiency panel performance of the 80 facilities is shown in (Table 1).

The score of the 80 TB microscopy sites for the 10 TB smears' proficiency panel is shown in (Table 2).

Table 2 shows that slide 4 was negative TB sputum in most facilities; 76 out of 80 (95%) responded to it correctly. The highest quantification error (QE) was reported for slide 6 which was 3+, and 46 (58%) facilities reported quantification error for this specific panel (Table 2). The highest false negative was observed from slide 10 which was 2+ in TB bacteria density.

### 3.2. On-Site Assessment of TB Laboratories' Performance in Liberia

The other EQA method for sputum microscopy included in our study was the on-site assessment of TB smear microscopy laboratories. The assessment was conducted with standard check. The assessment included availability of adequate laboratory space, laboratory safety, availability of trained staff, laboratory standard operating procedures (SOPS), laboratory equipment, and supplies. Although 107 functional TB smear microscopy laboratories in Liberia were included in the assessment, only 93 laboratories were visited and assessed in our study (Table 3).

EQA participation, laboratory reporting, and documentation are crucial components of TB laboratory service quality management and were included in the assessment (Table 4).

An assessment component of the performance of the TB microscopy laboratories, laboratory safety, was also included as part of the on-site assessment (Table 5).

### 3.3. Laboratory TB Case Detection

**Figure 1:**
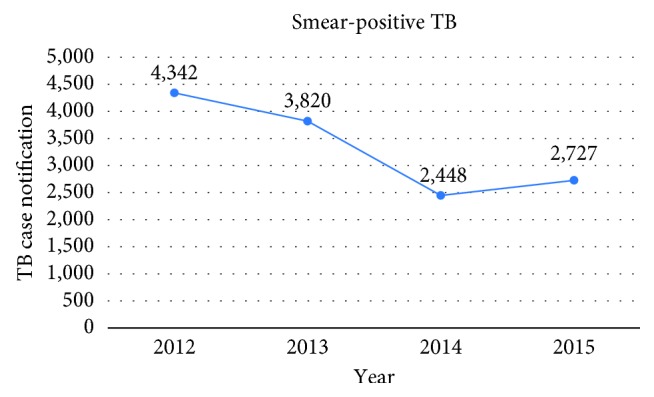
Notified TB smear-positive cases (2012–2015).

**Figure 2:**
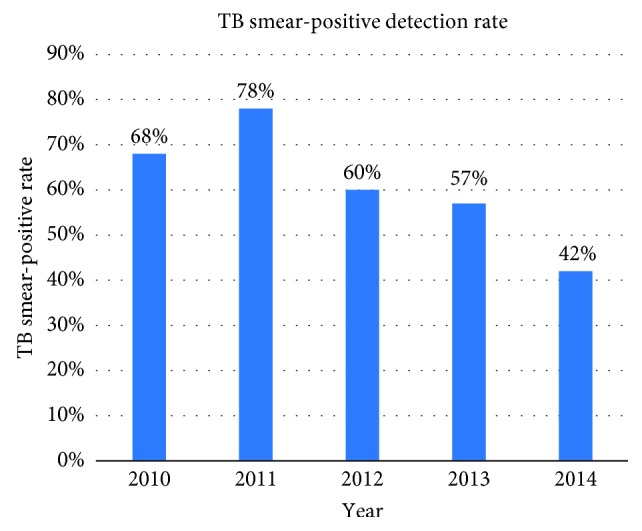
TB smear case detection % (2010–2015).

Laboratory case detection of TB plays a pivotal role in the control of TB. Sputum microscopy is one of the oldest and most affordable laboratory tests. This study included four years' (2012–2015) number and percentage of TB smear notified in Liberia (Figures 1 and 2). The TB notification data were used to show the performance of TB microscopy in Liberia.

## 4. Discussion

The prevalence of TB in Liberia is increasing from year to year. The prevalence was 341/100,000 population in 1990 and became 446/100,000 population in 2013 [5]. Despite this, the TB laboratory performance of Liberia has not improved for years with sputum microscopy being the only means of TB laboratory diagnosis till 2012. TB Solid Culture and DST Laboratory was initiated for the first time in 2012 and was interrupted during the 2014-2015 outbreak of Ebola virus disease (EVD). Liberia adopted a four-module GeneXpert technology for testing MTB/RIF assay in 2013 with two machines installed at the TB annex hospital and the Ghanta TB/leprosy rehabilitation centre.

Findings indicated a poor performance on EQA. Similar EQA poor performance was also reported in other studies in developing countries [12, 13]. Only two facility laboratories scored 100% in the TB sputum microscopy proficiency testing. Major errors include high false positive (HFP), low false positive (LFP), and high false negative (HFN). HFP is when a participating laboratory reports actual negative sputum as 2+ or 3+ TB bacilli load. HFN, on the contrary, is erroneously reporting a TB bacilli load of 1+, 2+, or 3+ as negative. Reporting of HFP error is harmful since a very toxic anti-TB drug will be administered to the patient for TB infection. Nevertheless, reporting an HFN error will mislead the clinician and he/she will be prevented from prescribing the drugs, and hence the patient will suffer from the disease. The patient will also spread the disease to the general community. Minor errors have similar consequences as the major errors. Major and minor errors are associated with TB microscopy laboratory staff's lack of knowledge on the morphology of stained TB bacilli. This can be improved by formally training the staff as well as engaging the laboratory to practise internal quality-control exercises and participate in regular EQA schemes. Regular refresher training on TB morphology and grading of the bacilli load should be provided to TB laboratory staff.

The result from the on-site assessment of TB microscopy sites indicated that in 40% of the TB microscopy laboratories assessed in our study, the TB testing was conducted by laboratory aids who were not formally trained in laboratory science. They only had on-the-job training to assist in laboratory activities. This might have significantly compromised the quality of TB service provided at these facilities and many patients may be misdiagnosed. The Ministry of Health of Liberia should advocate training of laboratory technologists to fill the gap in trained manpower for laboratory testing.

A separate area for TB work and a separate table for TB specimen processing were not available in 59% and 34% of the laboratories assessed, respectively. The assessment finding indicates that the standards for TB microscopy testing laboratories were not met during the construction of the health facilities. In a study conducted in a different resource-constrained setting by Linda et al. in 2011, similar findings were reported [6]. The assessment findings also indicated frequent stock-out of TB slides, slide boxes, staining reagents, and specimen cups during the assessment year.

Availability of SOPs, participation in EQA, and timely analysis and reporting of sputum microscopy results were also considered during the assessment of the TB laboratories. Eighty-eight (95%) laboratories did not have SOPs. Only job aids were available, which did not show the detail of testing procedures. Regarding the EQA, 64 (69%) of the assessed laboratories did not keep slides for blind rechecking by the national TB programme. Blind rechecking of TB slides is one of the EQA methods for TB where slides reported by the laboratory are rechecked by an external body. This is one of the critical gaps identified in the TB laboratories in Liberia. It was also noted during the assessment that 85 (95%) of the laboratories failed to take EQA corrective measures for performance deficiencies. A similar study conducted in Ghana [14] indicated that SOPs approved by the national TB programme were available in all TB microscopy laboratories serving as references. Our study finding also indicated that 96% of the assessed laboratories conducted same-day analyses of patients' TB sputum specimens and timely reporting to physicians. Staining reagent containers were also properly labelled, showing the date of preparation and expiration. The findings of this study conducted in Ghana were different from our study findings [6, 14].

Our study also indicated that TB microscopy testing was conducted by laboratory aids in most of the health centres at the TB microscopy sites that were assessed. The laboratory aids did not receive formal training, and the results delivered from these sites may be compromised. Assessments and proficiency panels were not provided in 14 of the 107 laboratories as there were no trained staff members to conduct the test. The Ministry of Health should consider the absorption of new graduates from different colleges by allocating enough budget. Some of the staff members also have no definite employment. This challenge could be overcome through formalisation of employment of volunteers and recruitment of additional healthcare staff to provide sustainable services. Including TB in preservice curricula and strengthening in-service training will solve the human resource problem and enhance the quality of TB service provided by the national TB programme of Liberia. In a study conducted in Uganda, in order to improve the workflow, task-shifting through training of a cadre of lay health workers to perform simple tasks like collecting sputum and initial patient registration was implemented [15].

Because of the high risk of TB infection to laboratory staff, the laboratory has to be well ventilated with biosafety hood, waste disposal, and safety procedures in place. Of the laboratories assessed, 20 (22%) did not have running water and 26 (28%) did not have waste containers with lid covers. In addition, of the 93 laboratories, 22 (34%) were not well ventilated. Only 2% of the 93 laboratories assessed in our study had biosafety hoods for sputum processing. Similar findings were indicated in studies conducted in developing countries [6, 7]. This situation is very dangerous as the aerosols generated in the sputum specimen processing may be accumulated in the laboratory and inhaled by the laboratory staff. A lack of safety practices and procedures was identified in this assessment.

Notified smear-positive cases of TB were 3820 in 2013 and dropped to 1664 during the heightened outbreak of EVD in Liberia. The TB case notification data of this study indicated a sharp drop in TB cases notified in 2014 due to the EVD outbreak. In 2015, the health facilities were reopened and the basic services were restored—including the TB diagnosis and treatment. As a result, there was an increase in notified cases of smear-positive TB. The case detection rate was as low as 42% in 2014 compared to 57% in 2013. This was far lower than the Global WHO End TB targets and strategies aimed at a 95% reduction in TB deaths and a 90% reduction in the TB incidence rate [16]. Even the TB detection rate of above 70% of the 2015 millennium development goal targets were not met [4, 6, 16]. The authors of this study; Desta et al. [4], stated the reason for not meeting the target in their previously published paper: “The decline in 2014 and 2015 was attributed to high rate of loss to follow-up as result of the EVD outbreak.” Even though Sierra Leone was among the three EVD-affected countries in West Africa, the case detection rate was 60% and the TB treatment success rate was 86.7% in 2015 [17]. This shows that the TB programme was well functioning in Sierra Leone during the outbreak compared to Liberia. A similar study conducted on the TB burden in Nigeria indicated the TB prevalence among HIV-negative people as 27%, the TB incidence rate as 158 per 100,000 population, and the TB mortality as 39,933 per 100,000 population [18].

One major constraint identified as limiting the attainment of this target was the noninvolvement of the private sector in the TB control programme in Liberia. The target of 70% case detection could only be reached as long as DOTS programmes continue to expand geographically and active involvement of the private sector is achieved. According to the WHO TB global report of 2014, this target was not attained by most countries in Africa. A similar trend was observed in a study conducted in fragile states: Afghanistan, Democratic Republic of the Congo, Haiti, and Somalia. In all four of these countries, the treatment success rate was 81–90%. This indicated an increase in the treatment outcome. On the contrary, the case detection rate for the four countries was in the range of 39–62% which is insufficient compared to the global targets set during the study period. Even though these percentages did not meet the global target, it is evident that TB control programmes function in fragile states despite logistic and security challenges [19].

## 5. Limitation of the Study

Our study included the main aspects of TB laboratory quality of testing assessment elements. Few elements of the laboratory proficiency elements such as TB sputum microscopy laboratory test result turnaround time and blind rechecking of TB slides are not included in our study, and these are the limitations of the study.

## 6. Conclusion

Our study findings showed significant performance deficiencies in the laboratory service. Only 25% of the 80 TB sputum microscopy laboratories that participated in the proficiency panel testing scored the acceptable result. Besides this, a lack of well-trained staff, adequate space for sputum processing, proper safety materials, SOPs, and quality-control TB slides were observed in our study. A shortage of laboratory reagents, slides for sputum microscopy and slide boxes, and a lack of uninterrupted supply of electricity were some of the challenges identified in this study. In addition, national and global targets for TB case detection and notification were not reached in our study. Comprehensive strengthening of the TB laboratory network focusing on quality of TB laboratory supportive supervision, regular external quality assessment, stock monitoring, promotion of infection control measures, and increased health staffing levels at the health facilities are crucial. The National Leprosy and Tuberculosis Control Programme of Liberia should secure sustainable financial resources and allocate sufficient funds to strengthen the TB laboratory testing service of the country and hence meet national and global targets.

### 6.1. Recommendation

The NLTCP of Liberia developed the TB laboratory diagnostic algorithm in 2009. The programme should revise the diagnostic algorism to include new diagnostic techniques like GeneXpert and culture and drug susceptibility testing to make provision for multidrug-resistant TB. The NLTCP should develop policies on the standards of training required for personnel working in TB microscopy laboratories; minimum requirements for TB laboratories; and retention of TB laboratory professionals working in the TB laboratories.

## Figures and Tables

**Table 1:** TB microscopy testing laboratories proficiency panel performance results (*N* = 80).

Facility performance	Number and % of laboratories	Liberia NLTCP scale	WHO recommended scale
Poor	9 (11%)	0–55	<65
Fair	8 (10%)	60–65	65–70
Fairly good	19 (24%)	70–75	70–80
Good	25 (31%)	80–85	80–85
Very good	18 (23%)	90–95	85–95
Excellent	2 (2%)	100	>95
Total number	80 (100%)		

**Table 2:** Proficiency panel scores of TB microscopy sites (*N* = 80).

Proficiency slide number	Health facility TB smear microscopy proficiency scores for the 10 slides					
	Correct	QE	LFN	HFN	LFP	HFP
Slide 1 lab report	48	27	0	5	0	0
Slide 2 lab report	58	0	0	22	0	0
Slide 3 lab report	11	1	66	0	0	0
Slide 4 lab report	76	0	0	0	1	0
Slide 5 lab report	58	4	0	18	0	0
Slide 6 lab report	27	46	0	5	0	0
Slide 7 lab report	58	0	1	20	0	0
Slide 8 lab report	39	1	39	0	0	0
Slide 9 lab report	71	0	0	0	3	4
Slide 10 lab report	60	9	0	10	0	0

QE = quantification error, minor error; LFN = low false negative, minor error; LFP = low false positive, major error; HFP = high false positive, major error; HFN = high false negative, major error.

**Table 3:** Human resource and laboratory space at microscopy sites (*N* = 93).

Human resource and laboratory space for TB testing	Microscopy sites (laboratories) (*N* = 93)	
	Number	Frequency (%)
Minimum qualification of laboratory staff		
Laboratory technologist	11	12
Laboratory technician	29	31
Laboratory assistant	16	17
Laboratory aid	37	40
Presence of trained personnel for TB microscopy		
Yes	93	100
No	0	0
Availability of separate area for the TB work		
Yes	38	41
No	59	59
Separate table for TB specimen		
Yes	61	66
No	32	34
Availability of weighing balance		
Yes	23	25
No	70	75
Adequate specimen cup		
Yes	77	83
No	16	17
Adequate stock of slides		
Yes	74	80
No	19	20
Adequate supply of staining reagents		
Yes	46	49.5
No	47	50.5
Adequate number of staining equipment		
Yes	92	99
No	1	1
Adequate stock of slide boxes		
Yes	47	50.5
No	46	49.5

**Table 4:** EQA participation, documentation, and reporting for TB microscopy testing.

Factors related to documentation and reporting	Microscopy sites (laboratories) (*N* = 93)	
	Number	Frequency (%)
Availability of standard operating procedures (SOPs)		
Yes	5	5
No	88	95
Timely result reporting		
Yes	90	96
No	3	4
Quality-control slides provided by TB programme		
Yes	9	10
No	84	90
Slides kept for EQA (blind rechecking) by TB programme		
Yes	29	31
No	64	69
EQA performance problem		
Yes	7	8
No	86	92
EQA corrective action		
Yes	8	9
No	85	91

**Table 5:** Biosafety practice in microscopy testing laboratories in Liberia.

Biosafety factors	Microscopy sites (laboratories) (*N* = 93)	
	Number	Frequency (%)
Availability of running water supply		
Yes	20	22
No	73	78
Waste container with lid		
Yes	67	72
No	26	28
Waste disposal system in place		
Yes	83	89
No	10	11
Well-ventilated TB laboratory		
Yes	71	76
No	22	34
Availability of biosafety hood for sputum processing		
Yes	2	2
No	91	98

## Data Availability

The data used to support the findings of this study are available from the corresponding author upon request.
